# Primary Tumor Surgery for Patients with De Novo Stage IV Breast Cancer can Decrease Local Symptoms and Improve Quality of Life

**DOI:** 10.1245/s10434-019-08092-2

**Published:** 2020-01-22

**Authors:** Yiran Si, Peng Yuan, Nanlin Hu, Xue Wang, Jie Ju, Jiayu Wang, Fei Ma, Yang Luo, Pin Zhang, Qing Li, Binghe Xu

**Affiliations:** National Cancer Center/National Clinical Research Center for Cancer/Cancer Hospital, Chinese Academy of Medical Sciences and Peking Union Medical College, Beijing, 100021 China

**Keywords:** De novo stage IV breast cancer, Local symptoms, Primary tumor surgery, Quality of life, Survival

## Abstract

**Background:**

It was unknown whether surgery for primary tumor would affect the occurrence of local symptoms caused by tumor progression in patients with de novo stage IV breast cancer (BC). Our work attempted to probe the effect of local resection on controlling local symptoms and improving the quality of life in de novo stage IV BC patients.

**Methods:**

Our study included patients presenting with de novo stage IV BC at the Cancer Hospital of the Chinese Academy of Medical Sciences from January 2008 to December 2014. In this study, we defined a new term called “local progress/recurrence of symptoms” (LPRS) to refer to the local problems caused by tumor progression/recurrence. All the patients were grouped into surgery and non-surgery groups. The characteristics of the two groups were analyzed by Chi square and Fisher’s test. Univariate and multivariate Cox regression models were designed to evaluate independent prognostic factors.

**Results:**

This study contained 177 patients. The follow-up deadline was April 1, 2019. The median follow-up time was 33 months (range 1–135 months). In included patients, 77 (43.5%) underwent surgery for primary tumors. Primary tumor surgery could reduce the occurrence of LPRS (relative risk/risk ratio (RR = 0.440; 95% CI 0.227–0.852; *p *= 0.015)) and patients without LPRS had longer OS (45 months vs 29 months, *p *< 0.001). In addition, patients who had only one symptom had better OS than those who had two or three symptoms (*p *= 0.0175).

**Conclusions:**

The quality of life in patients with de novo stage IV breast cancer can be improved by reducing the incidence of local symptoms through primary tumor surgery.

**Electronic supplementary material:**

The online version of this article (10.1245/s10434-019-08092-2) contains supplementary material, which is available to authorized users.

Generally, 3–5% of breast cancer (BC) patients in China are initially diagnosed as de novo stage IV BC.^1^ The standard therapeutic approach was systemic therapy, especially chemotherapy.^2^ Along with improvement of systemic treatment, which had prominently prolonged survival of patients, the effect of primary tumor surgery on de novo stage IV BC has gradually become a hot topic.^3^

To date, several prospective studies have focused on whether local resection can improve prognosis of patients diagnosed with de novo stage IV BC,^4^^–^6 but the final results have not been consistent, and other retrospective analyses have also yielded controversial results in answer to this question.^7^^–^10 Aside from survival, no studies have focused on the effect of surgery on local symptoms caused by tumor progression/recurrence. For patients with de novo stage IV BC who do not receive local resection, primary tumor progression may lead to lesion ulceration and bleeding; metastatic lymph node compression leads to limb edema, and the pain caused by large lesions is intolerable. For those who adopt local resection, local symptoms still occur due to postoperative recurrence or lymph node metastasis. All the local symptoms mentioned above could affect patient quality of life to some extent. So, in our study, we defined a new term called “local progress/recurrence of symptoms” (LPRS) to summarize the local problems caused by tumor progression/recurrence in de novo stage IV BC.

Therefore, the purpose of our retrospective work was to explore the effect of primary tumor surgery on controlling the occurrence of LPRS and to discover the potential relationships between surgery for primary tumor, LPRS and survival in de novo stage IV BC patients.

## Methods

### Study Design

This retrospective study included women diagnosed with BC in the Cancer Hospital of the Chinese Academy of Medical Sciences between 2008 and 2014. Patients were recognized as having de novo stage IV BC according to the eighth edition of the American Joint Committee on Cancer (AJCC) staging manual. Our inclusion criteria were that patients had (1) a pathology report identified as BC; and (2) a diagnosis of BC and simultaneous detection of distant metastasis or distant metastasis were discovered within 3 months of diagnosis with BC. Patients were removed from the study that (1) had incomplete clinical information and follow-up information; (2) had a previous history of other malignancies at the same time; or (3) only had postoperative complications.

It is necessary to elaborate on the new term LPRS. This study defined LPRS as local symptoms caused by tumor progression/recurrence in de novo stage IV BC patients after local resection or in the process of systemic therapy without primary tumor resection. LPRS was considered to occur when any of the following three conditions occurred: (1) visible ulceration, bleeding, or discharge of secretions from the lesion; (2) edemas of the face, neck or upper limb due to enlarged lesions or lymph node compression which may lead to limb movement disorder, paresthesia or affect normal life; (3) moderate or severe pain in breast or chest wall due to tumor progression or recurrence, but excluding pain caused by tumor ulceration and bleeding or pain caused by edema. It should be noted that postoperative complications were not included in our definition of the term LPRS. Pain was evaluated according to the commonly used clinical digital grading method (NRS), facial expression evaluation scale, or chief complaint pain grading method (VRS).

### Study Populations

We focused on different local symptoms during the follow-up. Patients can be divided into two groups based on their number of symptoms. One group had only one symptom according to definition of LPRS, and the other group had two or more symptoms.

We also searched for clinicopathologic features, such as age at diagnosis, primary tumor surgery, menopausal status, family history, metastasis sites and numbers, tumor size, clinical N stage, hormone receptor expression, HER-2 expression, Ki-67, response to first-line chemotherapy, use of radiotherapy, time until the local problem began and patient status at the last follow-up. The eighth edition of the AJCC Tumor Staging System was used to assess the size of tumor.^11^ The hormone-positives were at least 1% positive, and HER-2 positives were those that had immunohistochemistry-reported HER-2 overexpression (2 + or 3 +) and in situ hybridization-reported HER-2 amplification.^12^ Response evaluation criteria in solid tumors (RECIST) (version 1.1) were used to estimate response to treatment. OS was evaluated via clinical review or telephonic interview from the time of diagnosis until the last follow-up (censored) or death due to any cause. In addition, local progression-free survival (LPFS) was defined as from the date of diagnosis to the occurrence of LPRS.

### Statistical Analysis

The characteristics and local symptoms of the surgery and non-surgery groups were analyzed via Chi square and Fisher’s test. OS and LPFS were examined by Kaplan–Meier. Univariate and multivariate Cox regression models were designed to evaluate prognostic factors. All the statistical analyses made use of SPSS software (IBM SPSS version 22; IBM Corp., NY, USA) and GraphPad Prism (version 7.0). All tests were two-sided using a significance level of 0.05.

## Results

### Clinicopathological Characteristics of Patients

**Fig. 1:**
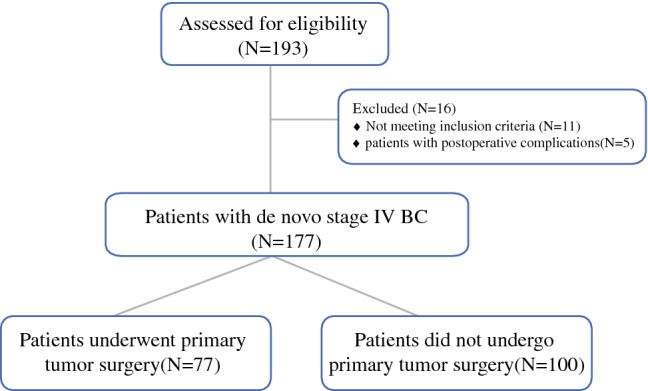
Flow diagram depicting a selection of patients

Patients presenting at the Cancer Hospital of the Chinese Academy of Medical Sciences between January 2008 and December 2014 who had de novo stage IV BC were included (*N* = 193). Excluding patients with incomplete clinical and follow-up data, with other malignancies, and with only postoperative complications, 177 patients were finally included (Fig. 1). The follow-up deadline was 1 April 2019. The median follow-up time was 33 months (range 1–135 months). Out of the 177 included patients, 77 (43.5%) patients underwent surgery for a primary tumor (surgery group), while 100 (56.5%) had no surgical treatment (non-surgery group). In the surgery group, 27/77 (35.0%) had LPRS, while 59/100 (59.0%) had LPRS in the non-surgery group. Furthermore, in the surgery group, 32/77 (41.6%) patients underwent modified radical mastectomy, 22/77 (28.6%) patients underwent simple resection, 4/77 (5.2%) patients underwent breast-conserving surgery, and 19/77 (26.7%) patients underwent palliative surgery. In addition, 68/77 (88.3%) patients in the surgery group accepted systemic treatment before surgery, 6 patients underwent surgery directly, and 3 patients received only endocrine therapy before surgery. The characteristics of patients are presented in Table 1.

**Table 1:** Comparison of clinical and tumor characteristics between patients who underwent surgery and those who did not

Characteristic	Non-surgery	Surgery	*p* value
Age at diagnosis			
≤ 45 years	11 (44.0%)	14 (56.0%)	0.196
> 45 years	89 (58.6%)	63 (41.4%)	
Menopausal status			
Premenopausal	48 (52.2%)	44 (47.8%)	0.288
Postmenopausal	52 (61.2%)	33 (38.8%)	
Family history			
No	81 (58.3%)	58 (41.7%)	0.461
Yes	19 (50.0%)	19 (50.0%)	
Bone metastasis			
No	44 (53.0%)	39 (47.0%)	0.448
Yes	56 (59.6%)	38 (40.4%)	
Viscera metastasis			
No	38 (52.1%)	35 (47.9%)	0.357
Yes	62 (59.6%)	42 (40.4%)	
Soft tissue and lymph node metastasis			
No	51 (49.5%)	52 (50.5%)	**0.032**
Yes	49 (66.2%)	25 (33.8%)	
Brain metastasis			
No	97 (56.1%)	76 (43.9%)	0.634
Yes	3 (75.0%)	1 (25.0%)	
No. of metastasis sites			
1–2	71 (51.1%)	68 (48.9%)	**0.006**
≥ 3	29 (76.3%)	9 (23.7%)	
Tumor size			
< 5 cm	51 (53.7%)	44 (46.3%)	0.612
≥ 5 cm	49 (59.8%)	33 (40.2%)	
Clinical N stage			
N0–N2	53 (55.3%)	43 (44.8%)	0.762
N3	47 (58.0%)	34 (42.0%)	
Differentiation degree			
High and medium	4 (5.6%)	67 (94.4%)	**0.016**
Low	96 (90.6%)	10 (9.4%)	
ER			
Negative	45 (54.2%)	38 (45.8%)	0.649
Positive	55 (58.5%)	39 (41.5%)	
PR			
Negative	63 (64.9%)	34 (35.1%)	**0.015**
Positive	37 (46.3%)	43 (53.7%)	
HER-2			
Negative	54 (62.8%)	32 (37.2%)	0.247
Positive	38 (51.4%)	36 (48.6%)	
Unknown	8 (47.1%)	9 (52.9%)	
Ki-67			
< 14%	28 (52.8%)	25 (47.2%)	0.62
≥ 14%	72 (58.1%)	52 (41.9%)	
Chemotherapy response			
CR + PR	46 (46.5%)	53 (53.5%)	**0.001**
SD + PD	48 (76.2%)	15 (23.8%)	
Unknown	6 (40.0%)	9 (60.0%)	
Radiotherapy			
No	93 (64.6%)	51 (35.4%)	**<0.001**
Yes	7 (21.2%)	26 (78.8%)	
LPRS			
Without	41 (45.1%)	50 (54.9%)	**0.002**
With	59 (68.6%)	27 (31.4%)	

Values in bold are significant at the 0.05 level

*CR* complete response, *PR* partial response, *PD* progression of disease, *SD* stable disease; *LPRS* local progression/recurrence of symptoms

### Primary Tumor Surgery Could Decrease the Occurrence of LPRS

From the results in Table 1, the patients in the non-surgery group were likely to have LPRS. The incidence rate of LPRS in the non-surgery group was 59.0%, while in the surgery group it was 35.0% (*p *= 0.002). To investigate whether surgery was a potential factor that affected the occurrence of LPRS, we analyzed the relevant clinicopathological features of the patients with LPRS and those without LPRS. The results showed that when controlling and adjusting for covariates, surgery for the primary tumor (RR = 0.440; 95% CI 0.227–0.852; *p *= 0.015) was the most important factor affecting the incidence of LPRS (Supplementary Table 1). Furthermore, ≥ 3 metastases (RR = 2.444; 95% CI 1.061–5.631; *p *= 0.036) and a high clinical T stage (T3–T4) (RR = 3.857; 95% CI 1.922–7.740; *p *< 0.001) were adverse factors for the occurrence of LPRS (Supplementary Table 1).

### Patients Without LPRS Lived Longer

**Fig. 2:**
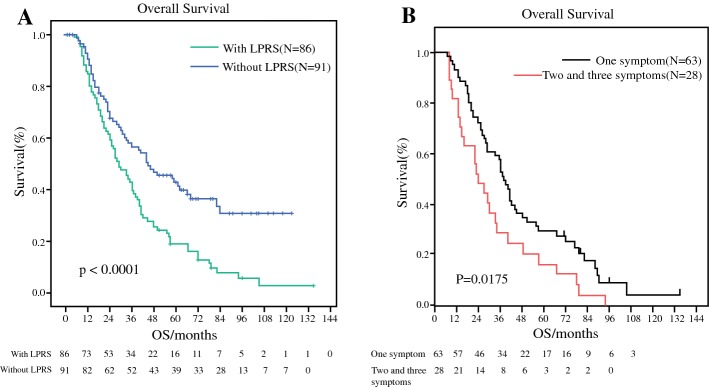
Kaplan–Meier curves estimate OS:** a** the OS between without LPRS vs. with LPRS (45 vs 29 months, *p* < 0.0001); ** b** the OS discrepancies in patients with only one symptom and patients with two and three symptoms (*p* = 0.0175)

We analyzed whether the occurrence of LPRS could affect survival. The results showed that patients with LPRS had obviously shorter OS than those without LPRS (29 months vs. 45 months) and there were significantly statistical differences between two groups (*p *< 0.0001) (Fig. 2a). We further discovered that patients with only one symptom had better OS than those with two or three symptoms (*p *= 0.0175) (Fig. 2b).

### Primary Tumor Surgery can Bring Survival Benefits

**Fig. 3:**
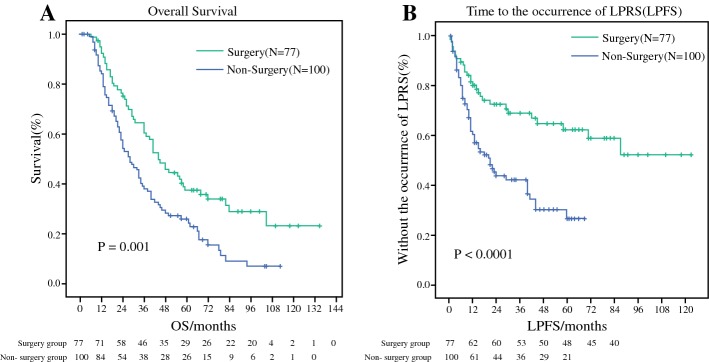
Primary tumor surgery can bring survival benefits and longer LPFS:** a** the OS between surgery group vs. non-surgery group (44 vs 28 months, *p* = 0.001).** b** The LPFS between surgery group vs. non-surgery group (42 vs 21 months, *p* < 0.0001)

In our study, patients who received surgery had better survival than patients without surgery (44 months vs 28 months, *p *= 0.001) (Fig. 3a). In addition to OS, we also compared the LPFS between the two groups. The final results showed that the LPFS in the surgery group was longer than in the non-surgery group (42 months vs 21 months, *p *< 0.0001) (Fig. 3b). Univariate and multivariate Cox hazard models indicated that the occurrence of LPRS (HR = 1.711; 95% CI 1.185–2.471; *p *= 0.004) and not undergoing surgery (HR = 0.663; 95% CI 0.453–0.972; *p *= 0.035) had independently adverse effects on the prognosis of patients with de novo stage IV BC (Table 2).

**Table 2:** Cox regression predicting mortality risk for patients with de novo stage IV BC (univariate and multivariate)

Characteristic	Univariate HR (95% CI)	*p* value	Multivariate HR (95% CI)	*p* value
Age	0.757 (0.479–1.196)	0.232		
Menopausal status	0.943 (0.605–1.471)	0.796		
No. of metastasis	1.704 (0.899–3.227)	0.102	1.586 (1.044–2.410)	**0.031**
Tumor size	1.056 (0.795–1.402)	0.708		
Differentiation degree	1.232 (0.877–1.731)	0.229		
PR	0.883 (0.482–1.620)	0.689		
ER	0.741 (0.417–1.316)	0.306	0.680 (0.479–0.966)	**0.031**
HER-2	1.044 (0.772–1.411)	0.781		
Ki-67	0.718 (0.470–1.095)	0.124		
Surgery or not	0.614 (0.388–0.971)	0.037	0.663 (0.453–0.972)	**0.035**
Chemotherapy response	1.288 (0.910–1.822)	0.153		
Radiotherapy	1.091 (0.617–1.929)	0.765		
With or without LPRS	1.649 (1.089–2.498)	0.018	1.711 (1.185–2.471)	**0.004**

Bold values represent which characteristics can predict mortality risk for patients with de novo stage IV BC by Cox regression

Based on the above research data, we could roughly draw the following conclusions: Local resection could reduce the incidence of LPRS; Patients without LPRS had longer OS; and in our study, surgery has been proved to bring survival benefits in de novo stage IV BC.

### The Next Treatment Preference for Patients Who Responded to First-Line Chemotherapy

In our study, 99/177 patients had effective first-line chemotherapy treatments (only PR, no CR in this study). Of these patients, 53 (53.5%) underwent surgery (surgery group), while 46 (46.5%) did not (non-surgery group). The results revealed that patients who did not have local resection after effective first-line treatment were prone to having LPRS (*p *= 0.045) (Table 3).

**Table 3:** Comparison of clinical and tumor characteristics between patients who underwent surgery and those who did not (chemotherapy effective patients)

Characteristic	Non-surgery	Surgery	*p* value
Age at diagnosis			
< 65 years	33 (44.6%)	41 (55.4%)	0.644
≥ 65 years	13 (52.0%)	12 (48.0%)	
Menopausal status			
Premenopausal	24 (42.9%)	32 (57.1%)	0.425
Postmenopausal	22 (51.2%)	21 (48.8%)	
Bone metastasis			
No	20 (42.6%)	27 (57.4%)	0.546
Yes	26 (50.0%)	26 (50.0%)	
Viscera metastasis			
No	10 (25.0%)	30 (75.0%)	**0.032**
Yes	36 (61.0%)	23 (39.0%)	
Soft tissue and lymph node metastasis			
No	19 (36.5%)	33 (63.5%)	**0.045**
Yes	27 (57.4%)	20 (42.6%)	
No. of metastasis sites			
1–2	25 (34.2%)	48 (65.8%)	<**0.001**
≥ 3	21 (80.8%)	5 (19.2%)	
Tumor size			
< 5 cm	25 (46.3%)	29 (53.7%)	0.802
≥ 5 cm	21 (46.7%)	24 (53.3%)	
Clinical N stage			
N0–N2	25 (49.0%)	26 (51.0%)	0.290
N3	21 (43.8%)	27 (56.2%)	
Differentiation degree			
High and medium	26 (39.4%)	40 (60.6%)	**0.017**
Low	20 (60.0%)	13 (39.4%)	
ER			
Negative	25 (48.1%)	27 (51.9%)	0.841
Positive	21 (44.7%)	26 (55.3%)	
PR			
Negative	19 (38.8%)	30 (61.2%)	0.160
Positive	27 (54.0%)	23 (46.0%)	
HER-2			
Negative	20 (50%)	20 (50%)	0.812
Positive	22 (44.9%)	27 (55.1%)	
Unknown	4 (40%)	6 (40%)	
Ki-67			
< 14%	6 (28.6%)	15 (71.4%)	0.085
≥ 14%	40 (51.3%)	38 (48.7%)	
Radiotherapy			
No	41 (54.7%)	34 (45.3%)	**0.005**
Yes	5 (20.8%)	19 (79.2%)	
LPRS			
Without	18 (36.0%)	32 (64.0%)	**0.045**
With	28 (57.1%)	21 (42.9%)	

Bold values represent that there were differences of characteristics between patients who had effective first-line chemotherapy treatments who underwent surgery versus patients who did not have surgery

**Fig. 4:**
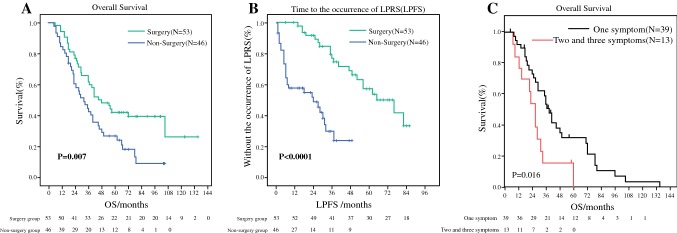
Kaplan–Meier curves estimate OS and LPFS in 99 patients who had effective treatment of first-line chemotherapy:** a** the OS between surgery group vs. non-surgery group (48 vs 32 months,* p* = 0.007).** b** The LPFS between surgery group vs. non-surgery group (66 vs 24 months,* p* < 0.0001).** c** The OS discrepancies in patients with only one symptom and patients with two and three symptoms (*p* = 0.016)

Then, we also did survival analysis. The result showed that patients who underwent surgery survived longer than those without surgery (48 months vs 32 months, *p *= 0.007) (Fig. 4a). We also examined the effect of surgery on LPRS. By comparing the LPFS between the two groups, the median time to the occurrence of LPRS in the non-surgery group was 24 months, and for the surgery group it was 66 months (*p* < 0.0001). LPFS in the surgery group was longer than in the non-surgery group for patients with effective first-line chemotherapy (Fig. 4b). Furthermore, the patients who had only one symptom showed better OS than those who had two or three symptoms (*p *= 0.016) (Fig. 4c).

### Further Screening for Which Types of Patients were Prone to Developing LPRS

**Fig. 5:**
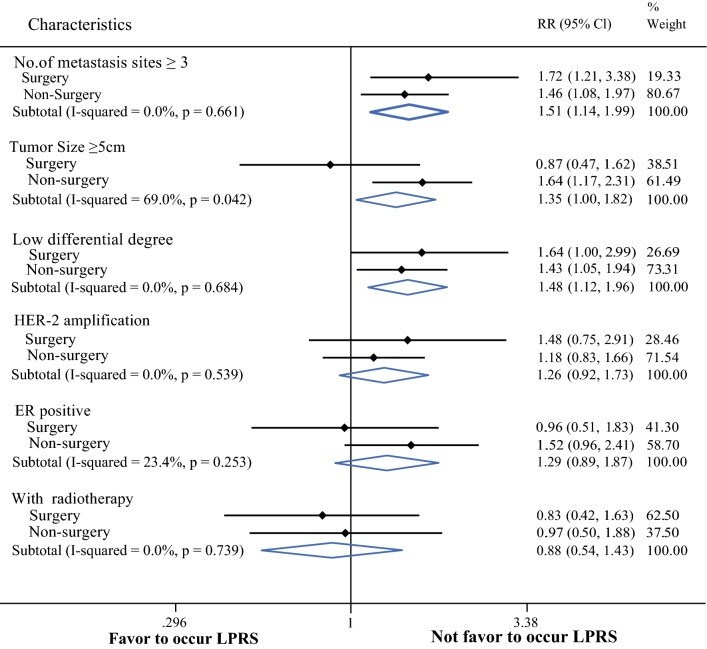
Forest plot to further clarify which kinds of patients are favor to occur LPRS

From the above analyses, it can be concluded that patients who were prone to developing LPRS had no survival advantage. Next, we conducted an exploratory stratification analysis of some important clinical features to help clinicians further clarify which kinds of patients were prone to developing LPRS. From the forest plot, patients with ≥ 3 metastases were more likely to have LPRS, regardless of whether they were in the surgery or non-surgery group (RR = 1.51; 95% CI 1.14–1.99; *p *= 0.661). While the degree of differentiation was lower, patients in the non-surgery group were inclined to develop LPRS (RR = 1.43; 95% CI 1.05–1.94; *p *= 0.684). Patients with tumors ≥ 5 cm in size did not undergo local resection and were prone to developing LPRS (RR = 1.64; 95% CI 1.17–2.31; *p *= 0.042) (Fig. 5). Other important clinical and tumor features, such as HER-2 amplification, ER-positivity, and local radiotherapy, were not associated with a tendency to develop LPRS, regardless of surgery status.

## Discussion

Currently most prospective or retrospective studies focused on OS and distant progression-free survival (DFS). Few researches mentioned the local progress and none of the studies concentrated on the control of local symptoms by primary tumor surgery. At present, there have been three prospective studies on the local resection of de novo stage IV BC; the Tata study, published in India, only vaguely mentioned local progression-free survival at the end of the results, and it did not further describe the local symptoms.^4^ In the Turkish study, the final results only suggested that the proportion of lesion ulcerations in patients who did not receive local treatment was higher than that in patients receiving local treatment (11% vs 1%, *p *= 0.001), and there were no other relevant data.^5^ The recent Austrian ABCSG-28 POSITIVE study did not mention relevant local problems.^6^ Therefore, in the Discussion of the Turkish study, the authors mentioned that the role of local resection in the control of local symptoms was an unsolved issue worthy of examination and research. Therefore, in our retrospective study, we proposed the research term “local progress/recurrence of symptoms” (LPRS) to represent the local symptoms caused by tumor progression/recurrence. This is the first time that our attention has been focused on the local symptoms due to tumor progression/recurrence, which directly affects the quality of life of patients. Whether local resection would affect the occurrence of LPRS and further influence the quality of life in de novo stage IV BC patients was the main focus of the present study.

First, we found that occurrence of LPRS in the surgery group was clearly lower than in the non-surgery group (35.1% vs 59.0%, *p *= 0.002). The lower incidence rate of LPRS in the surgery group was mainly because primary tumor surgery reduced the tumor burden, which may lead to less recurrence and metastasis. Consistent with existing clinical practice, our study showed that patients in the surgery group were more inclined to have effective first-line chemotherapy treatment before surgery (53.5%). The efficacy of the first-line treatment reflected the sensitivity of the tumor to treatment, and patients with high sensitivity tended to achieve better therapeutic effects after surgery treatment. It was also worth noting that patients with three symptoms simultaneously appeared only in non-surgical group, but none in surgical group (*p *= 0.005). Therefore, this result further confirmed that patients who have undergone local surgical resection were less prone to occur local symptoms. Moreover, in most clinical practices, stage IV BC patients who underwent surgery might always been younger, healthier and had more modest tumors than patients who did not undergo surgery. These characteristics may have led to better survival in patients who underwent surgery.^7^,^13^ In our study, except for lymph node/soft tissue metastasis, number of metastases, and differentiation degree, no differences were found in patients’ characteristics between the two groups (such as age at diagnosis, N-stage, HR, HER-2, Ki-67 and menopause status). These findings suggested that the conditions, such as being younger, healthier, and having a more modest tumor were not essential and that maybe more patients could benefit from surgery. Furthermore, from our results, tumor burdens (such as lymph node and soft tissue metastasis and the number of metastases) may act as a significant part in the choice of surgery. More studies need to be conducted to confirm the influencing factors that lead to surgery.

Second, in this study we demonstrated potential relationships between surgery, the occurrence of LPRS and survival. The results proved that primary tumor surgery could decrease the incidence of LPRS (RR = 0.440; 95% CI 0.227–0.852; *p *= 0.015), patients without LPRS have longer OS (45 months vs 29 months, *p *< 0.001), and the surgery has been proved to bring survival benefits and longer LPFS in this study (OS: 44 months vs 28 months, *p *= 0.001; LPFS 42 months vs 21 months, *p *< 0.0001). The role of surgery in survival is consistent with the conclusions of previous studies.^7^,^14^^–^17 Furthermore, patients with only one symptom had better OS than those with more than one symptom (*p *= 0.0175). Considering all above results, we can boldly speculate that de novo stage IV BC patients who received surgery are less likely to occur LPRS. Primary tumor surgery and/or absence of LPRS both can prolong survival for de novo stage IV BC patients. Therefore, we confirmed that there was a close relationship among primary tumor surgery, the occurrence of LPRS and survival. The more symptoms, the worse the prognosis. Furthermore, the number of metastasis sites and the clinical T stage are risk factors for the occurrence of LPRS (RR_metastasis number_ = 2.444; 95% CI 1.061–5.631; *p *= 0.036; RR_T stage_ = 3.857; 95% CI 1.922–7.740; *p *<0.001) and the number of metastases was also an independent adverse factor for prognosis (HR = 1.586; 95% CI 1.044–2.410; *p *= 0.031). Thus, the number of metastases not only affects the occurrence of LPRS but also the patient’s prognosis.

In the last part of the present study, we tried to solve some clinical issues. To address the next treatment step for patients with effective first-line chemotherapy, we conducted an exploratory analysis of patients who were evaluated for CR or PR during first-line chemotherapy after 4–6 treatment cycles in our study population. Surprisingly, the results suggested that in de novo stage IV BC patients for whom first-line chemotherapy was effective, the choice of local resection can not only prolong survival, but also delay the occurrence of LPRS. Next, we performed an exploratory stratified analysis in particular subgroups to determine which types of patients were prone to develop LPRS. These exploratory results revealed that patients who underwent surgery had ≥ 3 metastases, had a tumor diameter ≥ 5 cm, and had a low degree of differentiation were prone to developing LPRS. Although this was only an exploratory stratified analysis, combined with the above findings, for patients with a larger tumor burden in de novo stage IV BC, the decision to perform surgery needs to be made relatively cautiously.

From the above results, we concluded that the occurrence of LPRS not only influenced survival but also affected the patient’s quality of life. Previous research has confirmed that local and systemic symptoms are important manifestations for quality of life in BC patients. Local symptoms and pain are more prominent in advanced BC patients.^18^ Furthermore, some studies have confirmed that quality of life can help predict prognosis in stage IV BC patients other than early patients.^19^,^20^ Therefore, LPRS could represent the quality of life in patients with de novo stage IV BC to some extent. Therefore, we can prove that primary tumor surgery can decrease the incidence of LPRS and help improve the quality of life in de novo stage IV BC patients.

## Conclusion

The results of this study provide some references for the options of treatment strategies for de novo stage IV BC and suggest possible methods for addressing some intractable clinical problems. This was a single-center, small sample, retrospective study and selection bias was inevitable. In the future, we expect to further explore the relationship between primary tumor surgery and local symptoms in de novo stage IV BC, so as to bring better quality of life to patients.

## Electronic supplementary material

Below is the link to the electronic supplementary material.

Supplementary material 1 (DOC 28 kb)

## Abbreviations

BC: Breast cancer
LPRS: Progress/recurrence symptoms
OS: Overall survival
LPFS: Local progression-free survival
AJCC: American Joint Committee on Cancer
CR: Complete response
PR: Partial response
