# Supplementary material for: Primary Tumor Surgery for Patients with De Novo Stage IV Breast Cancer can Decrease Local Symptoms and Improve Quality of Life
Source: Ann Surg Oncol. 2020 Jan 22;27(4):1025–33. doi: 10.1245/s10434-019-08092-2 (PMC7060161; doi:10.1245/s10434-019-08092-2)
Supplement: Supplementary file 1 — Supplementary material 1 (DOC 28 kb) [file 10434_2019_8092_MOESM1_ESM.doc]

**Supplement Table 1** Adjusting covariates for influencing factors of LPRS

| Characteristic | B | S.E. | Wald | Free Degree | P Value | Exp(B) | 95% C.IEXP(B) | |
| --- | --- | --- | --- | --- | --- | --- | --- | --- |
|  |  |  |  |  |  |  | Lower | Upper |
| Primary Tumor Surgery | -.821 | .337 | 5.923 | 1 | .015 | .440 | .227 | .852 |
| No. of Metastasis | .894 | .426 | 4.405 | 1 | .036 | 2.444 | 1.061 | 5.631 |
| Clinical T Stage | 1.350 | .355 | 14.431 | 1 | .000 | 3.857 | 1.922 | 7.740 |
